# An evaluation of factors predicting long-term response to thalidomide in 234 patients with relapsed or resistant multiple myeloma

**DOI:** 10.1038/sj.bjc.6602225

**Published:** 2004-11-02

**Authors:** I Hus, A Dmoszynska, J Manko, M Hus, D Jawniak, M Soroka-Wojtaszko, A Hellmann, H Ciepluch, A Skotnicki, T Wolska-Smolen, K Sulek, T Robak, L Konopka, J Kloczko

**Affiliations:** 1 Department of Haematooncology, Medical University of Lublin, Poland; 2 Department of Haematology, Medical University of Gdansk, Poland; 3 Department of Haematology, Collegium Medicum Jagiellonian University, Cracow, Poland; 4 Department of Haematology CSK WAM, Warsaw, Poland; 5 Department of Haematology Medical University of Lodz, Poland; 6 Department of Haematology and Transfusion Medicine, Warsaw, Poland; 7 Department of Haematology Medical University of Bialystok, Poland

**Keywords:** multiple myeloma, thalidomide, pretreatment parameters, long-term response

## Abstract

The aim of this study was to assess the prognostic value of pretreatment clinical and laboratory parameters in refractory or relapsed multiple myeloma (MM) patients who have a long-term response to thalidomide (THAL), lasting at least 18 months. The study was carried out on 234 patients who received THAL for relapsed/refractory myeloma. Out of the 234 patients, 129 patients (55.1%) responded to THAL with a mean response duration of 11.9 months (ranging from 1 to 48) and an overall survival rate of 20.3 months (ranging 1–55 months). In 64 patients (27.4% of the whole group), the response to THAL lasted ⩾18 months with a mean response lasting 24 months. Statistical analysis of the group of nonresponders and patients with long-term response to THAL showed a significantly higher serum albumin level (*P*=0.0003) and haemoglobin level (*P*=0.05), as well as a lower *β*2 microglobulin (*β*2M) (*P*=0.022), LDH (*P*=0.045) serum level in patients with long-term response. In this study, the LDH and serum albumin level were predictors for response to THAL therapy. The *β*2M serum level was not a predictor for response to THAL. The albumin serum level was the best parameter distinguishing the group of patients with long-term response to THAL from the entire responding group (*P*=0.02).

Multiple myeloma (MM) is a plasma cell malignancy characterised by the accumulation of long-lasting plasma cells in the bone marrow and/or extramedullar sites. Conventional chemotherapy produces an overall response rate of 40–60% with a median survival of about 3.5 years. Aggressive, high-dose chemotherapy, bone marrow transplantation and intensive supportive care allowed for an increase in survival of up to 4–6 years. However, patients with primary resistant disease and those who relapse after a response have a median survival duration of 15 and 12 months, respectively. The disease still remains incurable and almost all patients eventually relapse and become resistant to cytostatics. New treatments have recently been developed to overcome drug resistance, which target the MM cell, the MM cell–host interaction and the bone marrow (BM) microenvironment. Thalidomide (THAL) and its immunomodulatory derivatives are examples of such agents that target the tumour cell in its BM milieu, and which make possible responses even in refractory or relapsed MM. THAL, used as a sedative drug in the 1960s, was withdrawn from clinical use because of its severe teratogenicity but has been reintroduced because of its immunomodulating and antiangiogenic properties (Rajkumar and Witzig, 2000). The exact mechanism of THAL action on MM is still unclear. Some postulated effects of THAL are: the inhibition of proangiogenic cytokines, such as vascular endothelial growth factor (VEGF), basic fibroblastic growth factor (bFGF) (Dmoszynska *et al*, 2000; Kyle and Rajkumar, 2001) and the downregulation of the secretion of proinflammatory cytokines such as interleukin-6 (IL-6), tumor necrosis factor (TNF) (Moreira *et al*, 1993; Turk *et al*, 1996); upregulation of adhesion molecules (Geitz *et al*, 1996) and the stimulation of cytotoxic T-cell proliferation and the secretion of interferon-*γ* and interleukin-2 (Haslett *et al*, 1998). First reports concerning the application of THAL in MM were published in 1999 by Singhal *et al* (1999). Since then, the efficacy of THAL in refractory or relapsed MM patients was confirmed by many other authors (Juliusson *et al*, 2000; Kneller *et al*, 2000; Barlogie *et al*, 2001a, 2001b; Hus *et al*, 2001; Kumar *et al*, 2003) with a response rate ranging from 32 to 64%. However, many questions, such as that of the optimal dose, duration of therapy and the factors predicting response to THAL treatment are still only partially answered.

The aim of this study was to assess the prognostic value of pretreatment clinical and laboratory parameters in refractory or relapsed MM patients with a long-term response to THAL, lasting at least 18 months.

## MATERIALS AND METHODS

### Patients

The study group comprised 234 patients (117 women and 117 men) with an average age at 59.6 years (ranging from 19 to 87 years) who received THAL for relapsed or refractory myeloma in eight Polish centres between March 1999 and October 2003. All patients signed an informed consent approved by the Local Ethics Committee and in agreement with STEPS (the System for Thalidomide Education and Prescribing Safety Programme). Thalidomide was supplied in 100 mg tablets by Gruenenthal GmbH (Aachen, Germany). The dosage was started at 200 mg daily, and increased as tolerated by 100 mg every week to a final dose of 400 mg daily after 3 weeks.

After reports concerning the synergy between THAL and dexamethasone (Dex) were published, Dex was added to THAL for newly included patients in a dose of 40 mg daily, divided into two doses for 4 days per month and 78 patients were treated with this protocol. There was no prior THAL monotherapy in this group.There were no statistical differences in the clinical and laboratory parameters among those patients on THAL monotherapy and those on the combated treatment.

The treatment was continued until either the disease progressed or adverse reactions ⩾ grade 3 according to WHO occured.

The pretreatment and monthly follow-up evaluation included a full blood count, renal and liver function tests: the serum levels of immunoglobulins, *β*2-microglobulin (*β*2M), lactate dehydrogenase, C-reactive protein and Bence–Jones protein in urine; serum protein electrophoresis and serum and urine light chains. There was also a monthly neurological examination.

The response to the treatment was evaluated every 4 weeks in the first 6–8 months of therapy. The criteria of complete remission were total disappearance of the serum monoclonal protein and/or a 90% decrease in the baseline value of the 24-h urinary excretion of light chain protein as well < 5% plasma cells in the bone marrow biopsy, normalisation of haemoglobin, albumin and calcium levels. Partial response was defined as a 50% or greater reduction in the pretreatment value of the M-protein in serum and urine and normalisation of serum calcium. Minor response was defined as a reduction between 25 and 50% of the pretreatment M-protein value. An increase of the M-protein concentration by 25% in relation to the lowest value found during the treatment, an increase in 24-hour urinary Bence–Jones protein excretion to more than 2.0 g day^−1^ and reappearance of the M-component in serum or urine were described as progression during the THAL therapy.

Patients were divided into three groups according to the response to THAL treatment: patients with no response (group A), patients with a response lasting between 3 and 18 months (group B) and patients with a long-term response, ⩾ 18 months (group C). The characteristics of the patient groups are presented in Table 1

**Table 1:** Patient characteristics before thalidomide (THAL) treatment: group A–nonresponders' group. Group B–patients with response for THAL treatment >3 months and < 18 months, group C–patients with response for THAL treatment ⩾18 months

	**Age median/range (years)**	**Sex**	**Stage according to Durie–Salmon**	**Type of paraprotein**	**Number of chemotherapy cycles before TAHL median/range**	**Number of chemotherapy lines before TAHL median/range**		
Group A	61	F-49	I-5	IgG-68	10	3		
	(19–87)	M-56	II-18	IgA-24	(3–49)	(1–5)		
			III-81	LCD-10				
			Solitare-1	IgD-2				
				NS-1				
Group B	60	F-34	I-3	IgG-47	10	3		
	(35–79)	M-31	II-14	IgA-17	(3–40)	(1–6)		
			III-48	LCD-1				
								
Group C	63	F-37	I–2	IgG-46	13	3		
	(32–79)	M-27	II–22	IgA-12	(4–63)	(1–6)		
			III-40	LCD-6				
								
	**B2 M (mg l^−1^) median/range**	**CRP (mg dl^−1^) median/range**	**LDH (U l^−1^) median/range**	**WBC (G l^−1^) median/range**	**HB (g dl^−1^) median/range**	**PLT (G l^−1^) median/range**	**Plasma cells in BM on biopsy (%) median/range**	**Serum albumins (g dl^−1^)median/range**
Group A	4.04	3.9	351	4.55	10.4	170	24	3.8
	(1.08–31.0)	(0.0–65.0)	(94.0–1500.0)	(1.8–18.5)	(5.0–15.9)	(11–506)	(3.0–83.0)	(1.9–5.3)
Group B	3.69	3.82	304	4.2	10.84	187	21	3.9
	(1.18–10.97)	(0.0–25.0)	(51.0–652.0)	(0.4–9.5)	(6.7–14.4)	(1.0–488.0)	(3.0–70.0)	(2.8–4.7)
Group C	3.2	3.72	293	3.85	11	183	24	4.1
	(1.4–17.7)	(0.0–19.3)	(46–923.0)	(1.8–13.0)	(7.5–14.6)	(27.0–817.0)	(1.0–90)	(2.96–4.85)

LCD=light chain disease; NS=nonsecretory.

.

An analysis of pretreatment parameters distinguishing patients with a long-term response to THAL (group C) from the other patient groups (B and C) was performed.

Toxicity and adverse events occurring during THAL therapy were evaluated according to the WHO grading system. The occurrence of grade ⩾2 WHO toxicity prompted a THAL dose reduction, whereas drug administration was to be stopped in case of ⩾3 WHO toxicity.

### Statistical analysis

The statistical significance of the differences observed between the three patient groups was determined by using the *U* Mann–Whitney and Spearman test. The effect was considered as statistically significant if the *P*-value of its corresponding test statistic was ⩽0.05.

## RESULTS

**Figure 1:**
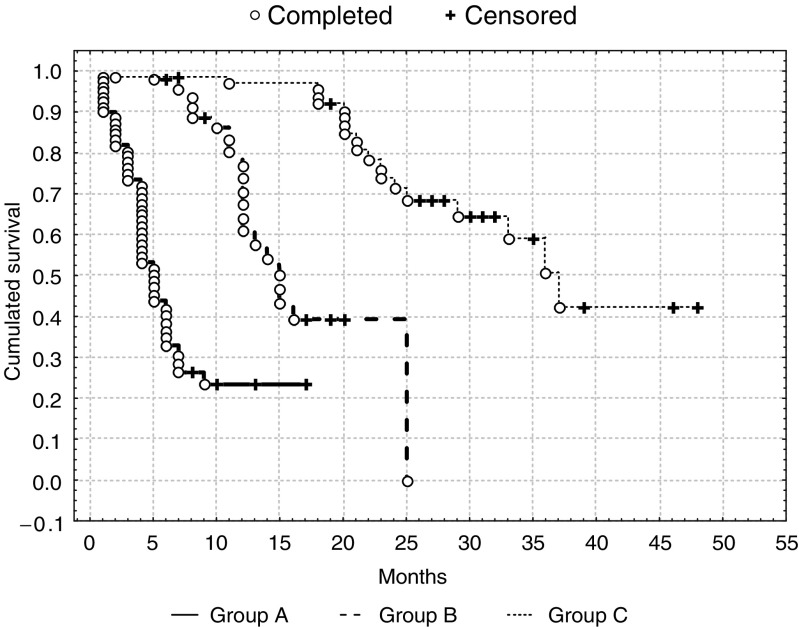
Event-free survival in patients group A, B and C (Test *F* Cox'*a*: *P*=0.00028 between group A and B; *P*=0.00005 between A and C; *P*=0.042 between B and C).

Out of 234 patients, 129 patients (55.1%) responded to THAL treatment with a mean response duration of 11.9 months (ranging from 1 to 48) and an overall survival rate of 20.3 months (range 1–55). In 64 patients (27.4% of the whole group), the response to THAL lasted ⩾18 months (group C) with a mean response lasting 24 months. In 105 patients (44.9%), there was no response (group A), while in 65 patients, the response lasted more than 3 months but less than 18 months (group B). The overall survival (OS) and event-free survival (EFS) rate in group A was 13.6 months (ranging from 1 to 38) and 4.2 months (1–15), respectively. Overall survival and EFS in group B was 22.7 months (range 7–54) and 11.37 months (3–24), respectively. The OS and EFS in group C was 29.1 months (ranging from 18 to 55) and 24 months ranging from (18–48). Differences in the OS and EFS were statistically significant (Figures 1

and 2

**Figure 2:**
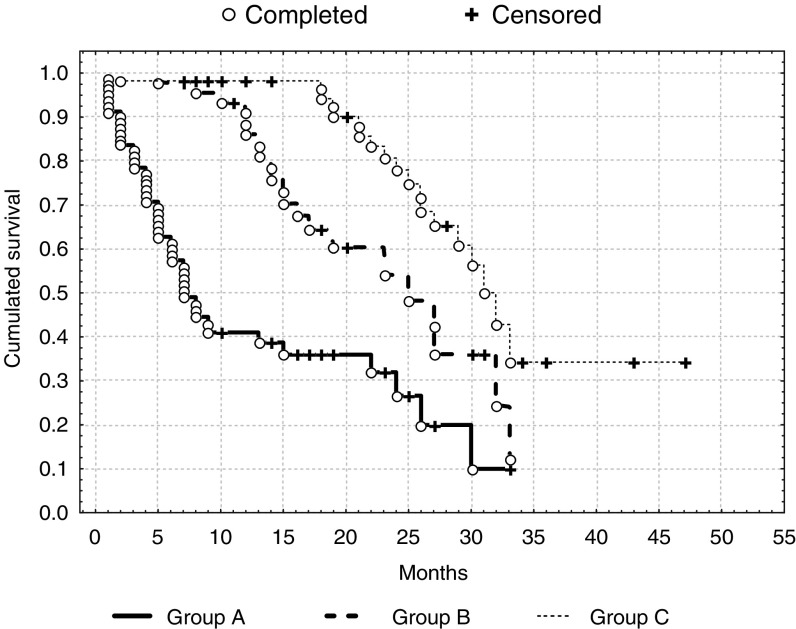
Overall survival in patients group A, B and C (Test F Cox'*a*: *P*=0.00032 between group A and B; *P*=0.00002 between A and C; *P*=0.022 between B and C).

).

In the entire patient group, the response rate to the combined treatment was slightly higher in patients receiving the THAL+Dex treatment than in patients on THAL monotherapy (59.3 *vs* 55.1%). However, the number of patients on the combined treatment or on THAL alone was similar in all three patient groups.

None of the estimated clinical parameters seemed to influence the duration of response to THAL therapy. The distribution of sex, the number of chemotherapy lines and cycles, number of refractory and relapsed patients, as well as the number of patients with prior autologous peripheral blood stem cell auto (autoPBSCT) was similar in the three groups of patients (Table 1).

**Figure 3:**
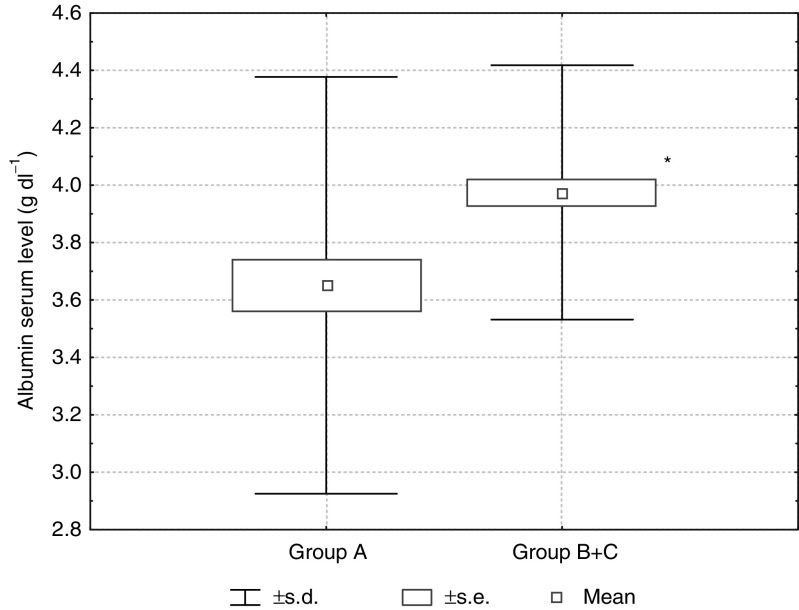
Albumin serum level in patients nonresponding (group A) and responding (group B+C) to THAL treatment (*P*=0.002).

**Figure 4:**
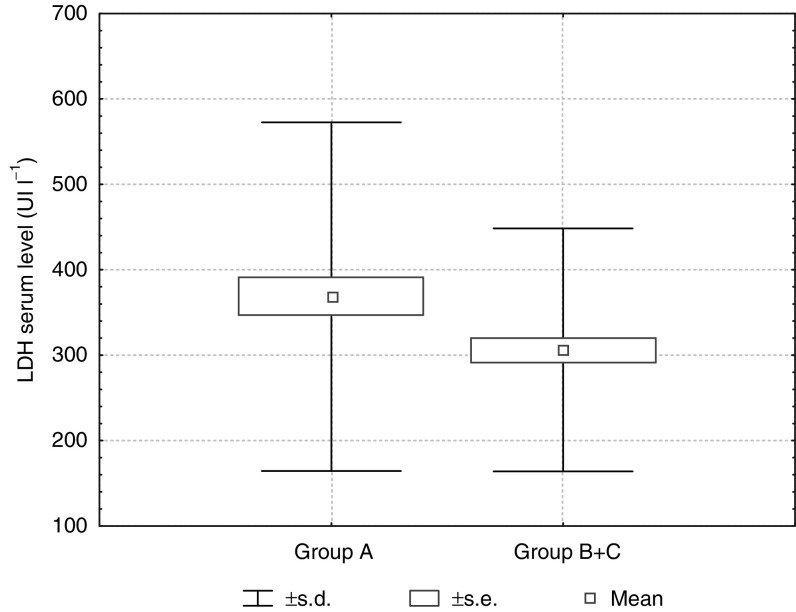
LDH serum activity in patients nonresponding (group A) and responding (group B+C) to THAL treatment (*P*=0.025).

Comparing the pretreatment laboratory parameters in patients responding to THAL and those not responding, statistically significant differences were found in the albumin serum levels (*P*=0.002) (Figure 3

), LDH (*P*=0.025) (Figure 4

) and the WBC count (*P*=0.049).

Statistical analysis of patients from groups A and C showed significantly higher serum levels of albumin (*P*=0.0003) and haemoglobin (*P*=0.05), as well as a lower level of *β*2M (*P*=0.022); and a decreased LDH serum activity (*P*=0.045) and WBC count (*P*=0.044) in group C (Table 1).

The only one significant difference between patient groups responding to THAL longer (group C) or shorter than 18 months (group B) was the higher pretreatment serum albumin level in group C (*P*=0.024) (Table 1).

**Figure 5:**
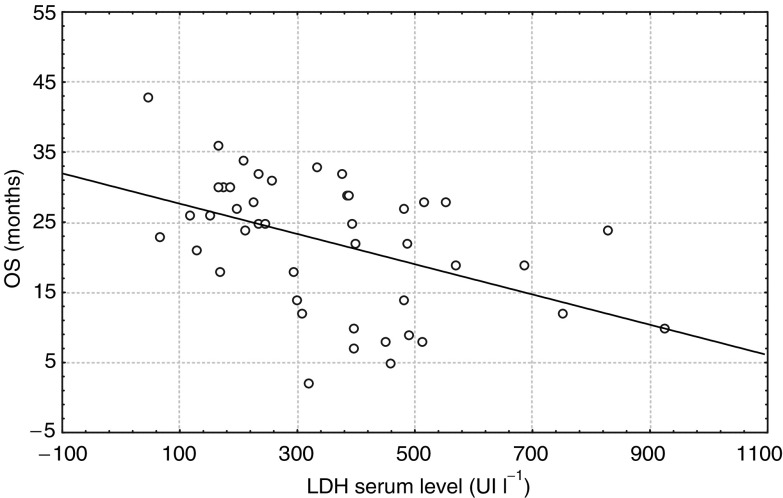
Correlation between LDH serum level and OS in patients with response to THAL lasting >18 months (group C) (*P*=0.0013).

An analysis of clinical and laboratory parameters and of the survival of patients in group C showed a statistically significant, negative correlation between the LDH serum level and the OS (Figure 5

).

**Figure 6:**
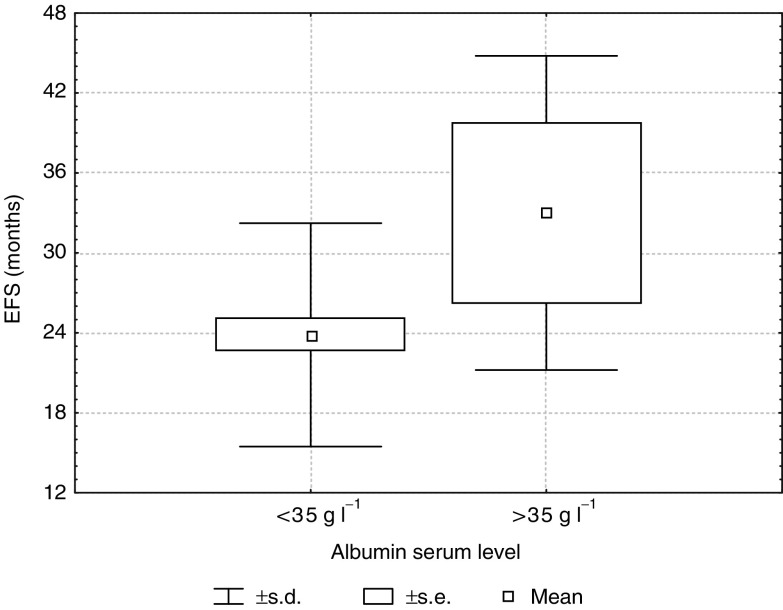
Event-free survival in patients with albumin serum level lower and higher than 35 g l^−1^ (*P*=0.003).

The serum albumin concentration was the only one parameter predicting a long-term response to THAL therapy. Moreover, a multivariate analysis showed that both the OS and EFS in the entire group of MM patients were influenced by a serum albumin level <35g l^−1^ (Tukey's test, *P*=0.009; *P*=0.003; respectively). In patients with a serum albumin level < 35g l^−1^ the mean EFS and OS were, respectively, 8.2±10.4 and 12.1±11.3 months. In patients with a serum albumin level >35 g l^−1^, the mean EFS and OS were, respectively, 14.8+10.4 and 17.6±9.4 months (Figures 6

and 7

**Figure 7:**
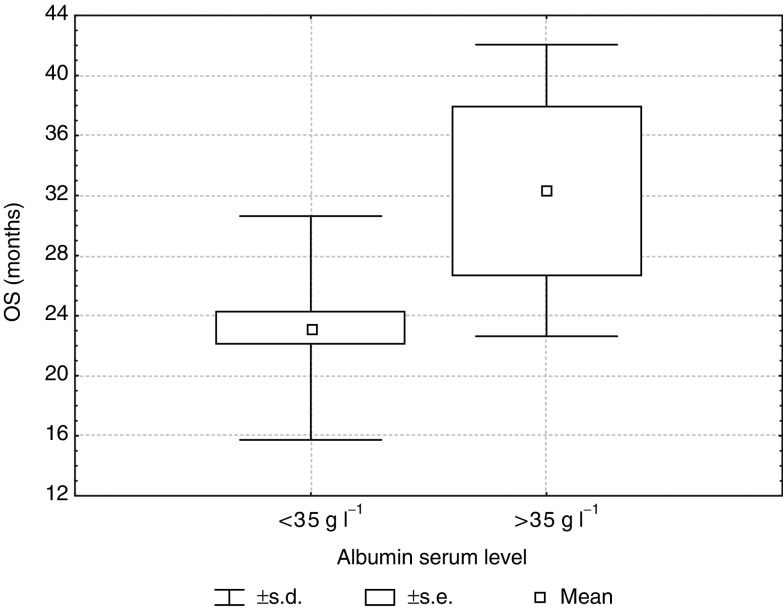
Overall survival in patients with albumin serum level lower and higher than 35 g l^−1^(*P*=0.009).

).

The adverse events connected with THAL therapy are listed in Table 2

**Table 2:** Adverse events during Thal treatment: group B: patients responding to Thal <18 months, group C: patients responding to Thal ⩾18 months

	**Toxicity according to WHO**			
	**Grade 1–2 (%)**		**Grade 3–4 (%)**	
**Adverse reaction**	**Group C**	**Group B**	**Group C**	**Group B**
Constipation	73.0	78.2	—	1.7
Fatigue	75.7	62.7	—	—
Somnolence	78.4	66.1	—	—
Skin rash	5.4	4	—	0.8 (1pt)
Neurological toxicity				
polyneuropathy	59.5	55	—	5.9
CNS cerebrovascular ischaemia	—	—	2.7 (1pt)	1.7 (2pts)
vertigo	—	2.5 (2 pts)	—	—
Deep vein thrombosis	—	—	2.7 (1 pt)	1.7 (2pts)
Cardiovascular toxicity				
Arrhythmia	13.5	10.2	—	—
Cardiac infarction	—	—	2.7 (1pt)	—
Chronic heart failure	—	1.5 (1 pt)	—	4.6
Leucopoenia	22.9	24.5	8.3	3.8

Pt=Patient.

. Constipation, somnolence and fatigue were observed in three-quarters of patients during THAL treatment. There were no significant differences between the frequency of the occurrence of adverse events in groups B and C. Seven patients (group B), developed polineuropathy (grade 3 according to WHO) resulting in the withdrawal of THAL treatment. Other adverse reactions causing the discontinuation of THAL therapy in single patients were: Deep vein thrombosis (DVT), cardiac arrhythmia, skin rash and cerebrovascular ischaemia. One patient died from myocardial infarction.

## DISCUSSION

Thalidomide, a glutamic acid derivative with antiangiogenic, immunomodulatory and proapoptotic properties, has been recently introduced as a salvage therapy for relapsed or refractory myeloma patients. It has been demonstrated to be highly effective as a single agent, not only in patients resistant to conventional chemotherapy but also in those relapsing after intensive chemotherapy with autologous stem cell transplantation, high-dose steroids or those who had been heavily pretreated (Juliusson *et al*, 2000). The results obtained in 234 patients, one of the largest reported groups of resistant or relapsed MM patients treated with THAL, as well as the results reported by several other authors, are presented in Table 3

**Table 3:** Thalidomide therapy in relapsed / refractory multiple myeloma

**Number of pts**	**Thal dose (mg d^-1^)**	**Follow-up period (months)**	**Response rate (%)**	**Comments**	
84	200–800	14.5	32	12-months OS 58%	Singhal *et al* (1999)
				12-months EFS 22%	
169	200–800	30	36	24-months OS 48%	Barlogie *et al* (2001)
				24-months EFS 20%	
83	50–800	18	66	18-months OS 40%	Yakoub-Agha *et al* (2002)
				18-months EFS 30%	
121	200–400	24	31.4	24-months OS 30%	Grosbois *et al* (2003)
				24-months EFS 15%	
75	200–800	26	28	18-months OS 48%	Mileshkin *et al* (2003)
				24-months PFS 18%	
191	200–400	39	56		Dmoszynska *et al* (2003)

.

The objective of our study was to identify the prognostic factors for long-term response (longer than 18 months), and the EFS and OS among clinical and laboratory parameters estimated before the start of THAL therapy.

*β*2M is a well-established prognostic factor in MM patients. A high serum *β*2M is a predictor of poor survival in patients treated with conventional chemotherapy (Rajkumar and Greipp, 1999). There is excellent correlation between serum *β*2M levels and myeloma tumour burden. It was shown that *β*2M was an independent predictor of both OS and EFS after transplantation for MM.

A statistical analysis of this study showed the lowest *β*2M serum level in patients with a response to THAL lasting more than 18 months, and the highest level in the nonresponders' group. This difference was statistically significant. The *β*2M serum level in patients with a response to THAL lasting between 3 and 18 months was higher than in patients with a long-term response, but this difference was not significant. So, in this study *β*2M was not a predictor of long-term response.

Another important laboratory parameter identified as a poor prognostic factor in MM patients is lactate dehydrogenase (LDH) serum activity. Elevated LDH concentration has been associated with an aggressive MM phenotype (Fonseca *et al*, 2001) and patients with a high LDH level have a shorter survival rate. We found that the LDH serum level was significantly higher in the nonresponding patients than in the responding ones. However, the mean level was similar in responding patients (group B and C) independent of the duration of response. The analysis of survival also showed a significant negative correlation between the OS and the LDH serum level in patients with a long response to THAL.

Of the parameters used in the Durie–Salmon staging system, only haemoglobin concentration was significantly higher in patients with a long-term response as compared to the nonresponding patients. In the nonresponders' group, low haemoglobin concentration correlates with the highest percentage of patients with the III clinical stage according to Durie–Salmon, as compared to the group of patients with a long-term response to THAL (77.5 *vs* 63%).

The albumin serum level, although not incorporated into the Durie–Salmon staging system, is also considered to be an important prognostic factor in MM. The albumin serum level was the highest in patients with a long-term response compared to the other groups, and the differences were statistically significant. In this study, the serum albumin level was the only one parameter distinguishing the group of patients with a long-term response to THAL treatment from the whole responding group (*P*=0.02). Moreover, in a multivariate analysis, a serum albumin level < 35 g dl^−1^ influenced the EFS and OS in the entire patient group.

An especially interesting case among our MM patients treated with THAL was the patient with an extremly long-term response to THAL therapy, achieved despite unfavourable prognostic factors at presentation, such as high serum *β*2M level, -13q deletion and MDR-1 gene expression. The patient did not respond to primary chemotherapy. It should be stressed that his serum albumin level was normal and the patient was succesfully treated with THAL for 36 months. Now, 14 months after THAL cessation, he is still in complete remission receiving no specific treatment. Moreover in this patient, THAL treatment resulted in the overcoming of the MDR (multidrug resistance) phenomenon.

The evaluation of baseline prognostic parameters predicting the response to THAL treatment, and the OS and EFS in refractory and relapsed MM patients was performed in previous studies by other authors. However, the patient groups were much less numerous and the observation period shorter.

In the first reported trial of THAL in patients with refractory myeloma, Singhal *et al* (1999) reported increased LDH levels, plasma-cell-labelling index (PCLI) and CRP levels as the predictors of a brief period of EFS, whereas low albumin levels, deletion of chromosome 13 and high numbers of plasma cells in BM were associated with relatively short OS. Barlogie *et al* (2001a), (2001b) found elevated CRP (>7 mg l^−1^), *β*2M levels (>3 mg l^−1^), high PCLI (>0.5%) and abnormal cytogenetics (deletion 13) to be a unfavourable prognostic factors for initial response to THAL and for EFS and OS in 169 MM patients treated with THAL.

Yakoub-Agha *et al* (2002) reported that pre-THAL features associated with a poor chance of OS and EFS included an IgA isotype, a platelet count <80 G/L and a serum albumin level <3 g l^−1^. In the prospective study of advanced MM treated with THAL, Grosbois *et al* (2003) found that the best factors for a good response were the status at inclusion (refractory or relapsed), a *β*2M level and platelet count. A multivariate analysis of the OS of relapsed or refractory MM patients demonstrated that advanced age (exceeding 65 years) raised the serum level of LDH and also the concentration of creatinine predicted inferior outcomes (Mileshkin *et al*, 2003).

Since an antiangiogenic effect is postulated as one of the most important mechanisms of THAL action pretreatment, angiogenesis was evaluated as a predictor of response. Cheng *et al* (1999) found that a high pretreatment microvessel density (MVD) predicted a poor response to THAL therapy, but this needs confirmation in a larger study. Neben *et al* (2001) reported that high plasma bFGF concentration is associated with a better response to THAL in progressive MM. Dmoszynska *et al* (2001), (2002) found that the major response to THAL was observed in patients with the highest VEGF pretreatment level compared to patients with a minor response.

As for now, there are no generally approved factors predicting the response to THAL in MM patients. The most often postulated ones are PCLI, abnormal cytogenetics, *β*2M and CRP. Since PCLI and cytogenetics are usually not available in the standard practice setting outside of specialised centres, *β*2M and CRP have an independent prognostic importance for survival. The results of our study confirm the results obtained by Singhal *et al* (1999) and Yakoub-Agha *et al* (2002) that the albumin serum level is a useful parameter, determining the response to THAL treatment.

In previous studies, the synergistic effect of THAL and Dex, as well as the prevalence of the combined treatment over monotherapy was shown (Cavenagh and Oakervee, 2003). The response rate to THAL+Dex in previously treated MM patients was up to 48% (Cavenagh and Oakervee, 2003; Anagnostopoulos *et al*, 2003). A higher response rate (64%) was observed only in newly diagnosed or untreated patients (Rajkumar *et al*, 2002). Weber *et al* (1999) achieved a response for THAL+Dex in 35% of patients previously resistant to THAL or Dex alone. In our study, the combined treatment of THAL+Dex had an increased response rate of 59.3% compared to THAL alone (55.1%). Although the number of patients on the combinated treatment or on THAL alone was similar in all the groups, the addition of Dex did not influence the duration of the response.

Several authors postulated a dependence between a cumulative dose of THAL administrated during the first 3 months of treatment and the clinical outcome. Barlogie *et al* (2001a), (2001b) evaluating patients treated with doses from 200 to 800 mg daily demonstrated that patients receiving a cumulative dose of more than 42 g of THAL in 3 had a higher rate of response (54 *vs* 21%) and a 2-year survival rate (63 *vs* 45%).

Neben *et al* (2002) noted that after 18 months of THAL treatment, patients tolerating the maximum THAL dose of 400 mg daily (a cumulative 3-month THAL dose of 31.8 g) had a predicted PFS and OS 15–20% higher when compared with patients with a dose of 200–400 mg (a cumulative 3-month THAL dose of 19.8 g). Other data show that responses lasting 15 months could be achieved with doses as low as 50 mg daily (Lee, 2002; Leleu *et al*, 2002).

In this study, in 3 months from the start of therapy, 85% of patients reached the dose of 400 mg daily (a cumulative dose of 34.4 g). This medium dose of THAL was effective in 129 patients (55.1%), and in 64 patients (29%) a long-term response was obtained lasting more than 18 months. It would be reasonable to reduce the drug dose to the lowest level that would still be effective. Unfortunately, the question of the minimal effective THAL dosage is still unresolved.

Although higher doses of THAL may be associated with a higher response rate, they may also be associated with a higher incidence of side effects and the discontinuation of therapy.

In doses of up to 400 mg daily, THAL is generally well tolerated. The most common side effects of the therapy are sedation, fatigue and constipation. They are generally mild and in these doses rarely lead to the discontinuation of treatment. A major potential adverse reaction connected with THAL is peripheral neuropathy, which can be irreversible. This generally occurs following a chronic use of THAL over a period of a month although reports regarding a relatively short-term use also exist. The correlation with the cumulative dose is unclear.

In this study, the incidence of polineuropathy was similar in groups of patients responding to THAL longer (group C) or shorter than 18 months (group B). In the majority of cases, it revealed itself within 12 months of therapy.

Neuropathy grade 3 according to WHO causing the discontinuation of therapy was observed in seven patients during the first 12 months of THAL treatment. Other serious side effects that may occur during THAL therapy are deep vein thrombosis (DVT) and CNS vascular ischaemia. The frequency of DVT and CNS vascular ischaemia was similar before (two patients, two patients, respectively) and after (one patient, two patients, respectively) 18 months of THAL therapy, respectively. Generally, long-term THAL therapy was safe and well tolerated.

The conclusion based on our results is that THAL as a single agent is effective in refractory and relapsed MM patients and that of various established MM prognostic factors, the pretreatment serum albumin level identified the group of patients with a long-term response to THAL therapy.

Taking into account the above conclusion, in the case of patients with a low pretreatment serum albumin level a decision concerning SCT should be considered immediately after achieving a response to THAL. It has been confirmed by many study groups that THAL as a single agent (Dmoszynska *et al*, 2000; Patriarca *et al*, 2003) or combined with VAD (Ahmad *et al*, 2002; Pitini *et al*, 2003) could be a suitable bridging regimen to PBSC collection and autologous SCT for VAD-refractory MM patients. Moreover, Ghobrial *et al* (2003) showed that THAL does not substantionally affect peripheral blood mobilisation or engraftment, which is in line with our observation.

For patients with low pretreatment level, other therapeutical options, such as a combination of THAL with chemotherapy or bortezomid, should be considered since such options can significantly increase the response rate. Total response rate to THAL combined with cyclophosphamide and Dex was 83 % (Garcia-Sanz *et al*, 2004). Other succesful combined regimens are T-VADdoxil (74%) (Zervas *et al*, 2004), THAL–melphalan (80%) (Offidani *et al*, 2003).

The majority of patients in our study responded to a medium dose of 400 mg. The long-term response rate was 50% of all patients responding to THAL therapy, and it was well tolerated by the majority of patients even in long-term therapy.

The efficiency of THAL and other new immunomodulatory drugs have opened up a new era in MM therapy. There is no doubt that THAL is the most active single agent used in relapsed or refractory MM patients in the last decade. In summarising this long-term observation, we can conclude that THAL is a relatively safe drug, which can be administered over a long period of time, offering many patients a longer life.

## References

[bib1] Anagnostopoulos A, Weber D, Rankin K, Delasalle K, Alexamnn R (2003) Thalidomide and dexamethasone for resistant multiple myeloma. Br J Haematolo 121: 768–771 10.1046/j.1365-2141.2003.04345.x 12780791

[bib2] Ahmad I, Islam T, Chanan -Khan A, Hahn T, Wentling D, Becker JL, McCarthy Jr PL, Alam AR (2002) Thalidomide as salvage therapy for VAD-refractory multiple myeloma prior to autologousPBSCT. Bone Marrow Transplant 27: 577–580 10.1038/sj.bmt.1703522 11979306

[bib3] Barlogie B, Desikan R, Eddlemon P, Spencer T, Zeldis J, Munshi N, Badros A, Zangari M, Anaissie E, Epstein J, Shaughnessy J, Ayers D, Spoon D, Tricot G (2001a) Extended survival in advanced andrefractory multiple myeloma after single-agent thalidomide: identification of prognostic factors in aphase 2 study of 169 patients. Blood 98: 492–494 11435324 10.1182/blood.v98.2.492

[bib4] Barlogie B, Tricot G, Anaissie E (2001b) Thalidomide in the management of multiple myeloma. Semin Oncol 28: 577–582 11740812 10.1016/s0093-7754(01)90027-2

[bib6] Cavenagh JD, Oakervee H (2003) Thalidomide in multiple myeloma- current status and future prospects. Br J Haematol 120: 18–26 12492572 10.1046/j.1365-2141.2003.03902.x

[bib7] Cheng D, Kini AR, Rodriguez J, Burt R, Peterson LC, Tray nor AE (1999) Microvascular density and cytotoxic T cell activation correlate with response to thalidomide therapy in myeloma patients. Blood 94(Suppl 1): 315a

[bib8] Dmoszynska A, Bojarska-Junak A, Domanski D, Rolnski J, Hus M, Soroka-Wojtaszko M (2002) Production of proangiogenic cytokines during thalidomide treatment of multiple myeloma. Leuk Lymph 43: 401–406 10.1080/10428190290006224 11999576

[bib9] Dmoszynska A, Hus M, Legiec W, Walter-Croneck A, Wach M (2000) Delayed stem celltransplantation in patients with relapsed or refractory multiple myeloma using lhalidomide as a preparative regimen. Blood 96(Suppl 1): 5345

[bib10] Dmoszynska A, Hus M, Soroka-Wojtaszko M, Mariko J, Jawniak D, Legiec VV, Grzasko N, Hellmann A, Ciepluch H, Baran W, Skotnicki A, Wolska-Smolen T, Sutek K, Borysewicz-Czajka T, Sawicki W, Robak T, Szmigielska A, Konopka L, Pszenna E, Szpila T, Kloczko J, Piszcz J, Zdziarska B (2003) Multicenter clinical study of thalidomide efficacv in patients with refractory and relapsed multiple myeloma. Hematology J 4(Suppl 1): 226

[bib11] Fonseca R, Conte G, Greipp PR (2001) Laboratory correlates in multiple mveloma: how useful for prognosis? Blood Rex 15: 97–102 10.1054/blre.2001.0154 11409909

[bib12] Garcia-Sanz R, Gonzalez-Poraz JR, Hernandez JM, Polo-Zarzuela M, Surreda A, Barrenetxea C, Palomera L, Lopez R, Grande-Garcia C, Alegre A, Vargas-Pabon M, Gutierez ON, Rodrigez JA, San Miguel JF (2004) The oral combination of thalidomide, cyclophosphamide and dexamethasone (ThaCyDex) is effective in relapsed/refractory multiple myeloma. Leukemia 18: 856–863 14973508 10.1038/sj.leu.2403322

[bib13] Geitz H, Handt S, Zvvingenberger K (1996) Thalidomide selectively modulates th density of cell surface molecules involved in the adhesion cascade. Immunopharmacology 31: 213–221 8861747 10.1016/0162-3109(95)00050-x

[bib14] Ghobrial IM, Dispenzieri A, Bundy KL, Gastineau DA, Rajkumar SV, Therneau TM, Lacv MQ, Witzig TE, Litzow MR, Christensen BR, Hayman S, Pribula CG, Gertz MA (2003) Effect of thalidomide on stem call collection and engraftment in patients with multiple myeloma. Bone Marrow Transplant 32: 587–592 12953131 10.1038/sj.bmt.1704173

[bib15] Grosbois B, Belissant P, Moreau P, Attal M, Voillat L, Muret P, Pegourie B, Tiab M, Berthou C, Duguet C (2003) Treatment of advanced multiple mveloma (MM) with thalidomide (THAL). Long term folow-up in a prospective study of 121 patients. Hematulogy 4(Suppl 1): 226

[bib16] Haslett PA, Corral LG, Albert M, Kaplan G (1998) Thalidomide costimulates primary human T lymphocytes, preferentially inducing proliferation, cytokine production and cytotoxic responses in the CD8+ subset. J Exp Med 187: 1885–1892 9607928 10.1084/jem.187.11.1885 PMC2212313

[bib17] Hus M, Dmoszyriska A, Soroka-Wojtaszko M, Jawniak D, Legiec W, Ciepluch H, Hellmann A, Wolska-Smoleh T, Skotnicki A, Manko J (2001) Thalidomide treatment of resistant or relapsed multiple mveloma patients. Hematologica 86: 404–408 11325647

[bib18] Juliusson G, Celsing F, Turesson I, Lenhoff S, Adriansson M, Malm C (2000) Frequent good partial remissions from thalidomide including best response ever in patients with advanced refractor), and relapsed myeloma. Br J Haemalol 109: 89–96 10.1046/j.1365-2141.2000.01983.x 10848786

[bib19] Kneller A, Raanani P, Hardan I, Avigdor A, Levi I, Berkowicv M, Ben-Bassat I (2000) Therapv with thalidomide in refractor) multiple myeloma patients–the revival of an old drug. Br J Haematol 108: 391–393 10691870 10.1046/j.1365-2141.2000.01835.x

[bib20] Kumar S, Gertz MA, Dispenzieri A, Lacy MQ, Geyer SM, Iturria NL, Fonesca R, Hayman SR, Lust JA, Kyle RA, Greipp PR, Witzig TE, Rajkumar SV (2003) Response rate, durability of response, and survival after thalidomide therapy for relapsed multiple mveloma. Mayo Clin Proc 78: 34–39 12528875 10.4065/78.1.34

[bib21] Kyle RA, Rajkumar V (2001) Therapeutic application of thalidomide in multiple myeloma. Semin Oncol 28: 583–587 11740813 10.1016/s0093-7754(01)90028-4

[bib22] Lee FC (2002) Second response to lower-dose thalidomide in a patient with multiple mveloma. Blood 99: 4248 12043696 10.1182/blood.v99.11.4248

[bib23] Leleu X, Magro L, Fawaz A, Banters F, Facon T, Yacoub-Agha I (2002) Efficacy of a low dose of thalidomide in advanced multiple myeloma. Blood 100: 1519–1520 12184283 10.1182/blood-2002-05-1527

[bib24] Mileshkin L, Biagi JJ, Mitchel P, Underhill C, Grigg A, Bell R, McKendrick J, Briggs P, Seymour JF, Lillie K, Smith JG, Zeldis JB, Prince HM (2003) Multicenter phase 2 trail of thalidomide in relapsed refractory multiple myeloma: adverse prognostic impact of advanced age. Blood 102: 69–77 12637329 10.1182/blood-2002-09-2846

[bib25] Moreira AL, Sampaio EP, Zmuidzinas A, Frindt P, Smidt KA, Kaplan G (1993) Thalidomide exerts its ihibitory action on tumor necrosis factor alpha by enhancing mRNA degradation. J Exp Med 177: 1675–1680 8496685 10.1084/jem.177.6.1675 PMC2191046

[bib26] Neben K, Moehler T, Egerer G, Kraemer A, Hillengass J, Benner A, Mo AD, Goldschmidt H (2001) High plasma basic fibroblast growth factor concentration is a^ociaied with response to ihalidomide in progressive multiple myeloma. Clin Cancer Res 7: 2675–2681 11555579

[bib27] Neben K, Moehler T, Benner A, Kraemer A, Egerer G, Ho AD, Goldschmidt H (2002) Dose- dependent effect of thalidomide on overall survival in relapsed multiple mveloma. Clin Cancer Res 8: 3377–3382 12429624

[bib28] Offidani M, Marconi M, Corvatta L, Olivieri A, Catarini VI, Leoni (2003) Thalidomide plus oral melphalan for advanced multiple myeloma: a phase II study. Haematologica 88: 1432–1433 14688003

[bib29] Patriarca F, Sperotto A, Prosdocimo S, Geromin A, Zaja F, Fanin R (2003) Thalidomide before autologous stem cell transplantation in VAD-refractorv multiple mveloma patients. Huematologica 88: 597–599 12745281

[bib30] Pitini V, Arrigo C, Aloi G, Micali C, La Gattuta G (2003) I halidomide as a salvage therapv for VAD-refractorv multiple myeloma prior to autologous PBSCT. Marrow Transplant 31: 1065 10.1038/sj.bmt.1704079 12774062

[bib31] Rajkumar SV, Greipp PR (1999) Prognostic factors in multiple myeloma. Hematol Oncol Clin N Am 13: 1295–1314 10.1016/s0889-8588(05)70128-3 10626152

[bib32] Rajkumar VS, Hayman S, Gertz MA, Dispenzieri A, Lacy MQ, Greipp PR, Geyer S, Iturria N, Fonseca R, Lust JA, Kyle R, Witzig TE (2002) Combination therapy with thalidomide plus dexamethasone for newly diagnosed myeloma. J Clin Oncol 20: 4319–4323 12409330 10.1200/JCO.2002.02.116

[bib33] Rajkumar SV, Witzig TE (2000) A review of angiogenesis and antiangiogenic therapy with thalidomide in multiple myeloma. Cancer Treat Rev 26: 351–362 11006136 10.1053/ctrv.2000.0188

[bib34] Singhal S, Mehta J, Desikan R, Roberson P, Eddelmon P, Munshi N, Anaissie E, Wilson C, Dhodapkar M, Zeddis J, Barlogie B (1999) Antitumor acmity of thalidomide in refractory multiple myeloma. N Engl J Med 341: 1565–1571 10564685 10.1056/NEJM199911183412102

[bib35] Turk BO, Jiang H, I.iu JO (1996) Binding of thalidomide to alpha I-acid glycoprotein may be involved in its inhibition of tumor necrosis factor alpha production. Proc Natl Acad Sci USA 93: 7552–7556 8755512 10.1073/pnas.93.15.7552 PMC38783

[bib36] Weber DM, Gavino M, Delasalle K, Rankin K, Giralt S, Alexanian R (1999) Thalidomide alone or with dexamethasone for multiple myeloma. Blood 94: 604a 10.1200/JCO.2003.03.139 12506164

[bib37] Yakoub-Agha I, Attal M, Dumontet C, Delannoy V, Moreau P, Berthou C, Lamy T, Grosbois B, Dauriac C, Dorvaux V, Bay JO, Monconduit M, Harousseau JL, Daguet C, Duhamel A, Facon T (2002) Thalidomide in patients with advanced multiple myeloma: a study of 83 patients-report of the intergroupe francophone du myelome (IFM). Hematology 7: 3:185–3:192 10.1038/sj.thj.6200175 12189564

[bib38] Zervas K, Dimopoulos A, Hatzicharissi E, Agruistopoulous A, Papaioannau M, Mitsouli Ch, Panagiotidis P, Korantzis J, Tzilianos M, Maniatis A (2004) Primary treatment of multiple myeloma with thalidomide, vincristine, liposomal doxorubicin and dexamethasone (T-VAD doxil): a phase II multicenter studv. Ann Oncol 15: 134–138 14679133 10.1093/annonc/mdh026

